# Enhanced Production of Chikungunya Virus-Like Particles Using a High-pH Adapted *Spodoptera frugiperda* Insect Cell Line

**DOI:** 10.1371/journal.pone.0094401

**Published:** 2014-04-08

**Authors:** James M. Wagner, J. David Pajerowski, Christopher L. Daniels, Patrick M. McHugh, Jessica A. Flynn, John W. Balliet, Danilo R. Casimiro, Shyamsundar Subramanian

**Affiliations:** Vaccine Research and Development, Merck Research Laboratories, Merck & Co., Inc., West Point, Pennsylvania, United States of America; Agency for Science, Technology and Research - Singapore Immunology Network, Singapore

## Abstract

Chikungunya virus-like particles (VLPs) have potential to be used as a prophylactic vaccine based on testing in multiple animal models and are currently being evaluated for human use in a Phase I clinical trial. The current method for producing these enveloped alphavirus VLPs by transient gene expression in mammalian cells presents challenges for scalable and robust industrial manufacturing, so the insect cell baculovirus expression vector system was evaluated as an alternative expression technology. Subsequent to recombinant baculovirus infection of *Sf*21 cells in standard culture media (pH 6.2–6.4), properly processed Chikungunya structural proteins were detected and assembled capsids were observed. However, an increase in culture pH to 6.6–6.8 was necessary to produce detectable concentrations of assembled VLPs. Since this elevated production pH exceeds the optimum for growth medium stability and *Sf*21 culture, medium modifications were made and a novel insect cell variant (*Sf*Basic) was derived by exposure of *Sf*21 to elevated culture pH for a prolonged period of time. The high-pH adapted *Sf*Basic insect cell line described herein is capable of maintaining normal cell growth into the typical mammalian cell culture pH range of 7.0–7.2 and produces 11-fold higher Chikungunya VLP yields relative to the parental *Sf*21 cell line. After scale-up into stirred tank bioreactors, *Sf*Basic derived VLPs were chromatographically purified and shown to be similar in size and structure to a VLP standard derived from transient gene expression in HEK293 cells. Total serum anti-Chikungunya IgG and neutralizing titers from guinea pigs vaccinated with *Sf*Basic derived VLPs or HEK293 derived VLPs were not significantly different with respect to production method, suggesting that this adapted insect cell line and production process could be useful for manufacturing Chikungunya VLPs for use as a vaccine. The adaptation of *Sf*21 to produce high levels of recombinant protein and VLPs in an elevated pH range may also have applications for other pH-sensitive protein or VLP targets.

## Introduction

Chikungunya virus (CHIKV) is an arbovirus (family *Togaviridae*, genus *Alphavirus*) spread by mosquitoes and capable of causing debilitating, long-term joint pain and arthralgia similar to Dengue [1], [2]. There is currently no CHIKV-specific therapeutic treatment or effective prophylactic vaccine [3], and the virus is associated with high rates of morbidity [4]. Periodic CHIKV outbreaks occur in Africa, southeast Asia, and the Indian Ocean islands, and spread to southern Europe was first reported in 2007 [5]. Outbreaks were documented in Cambodia, Papua New Guinea, and the Philippines in 2012 [6], and the first indigenous transmission in the Americas was reported in 2014 [7]. The recognized insect vector for CHIKV was historically *Aedes aegypti*, but a single point mutation in CHIKV has been associated with broadening of the geographical range of disease by increasing virus fitness for the alternative mosquito vector *Aedes albopictus* [8]. This potential for spread of the disease in new areas, including southern Europe, Australia, the Caribbean, and the Americas, has spurred increased research into methods for developing and producing an effective CHIKV vaccine [9], [10]. Many modalities have been evaluated or are currently under investigation, including an attenuated CHIKV vaccine [11], a formalin inactivated CHIKV vaccine [12], chimeric alphavirus vaccines [13], a consensus-based DNA vaccine [14], a Modified Vaccinia Ankara vector vaccine [15], an adenovirus vector vaccine [16], subunit vaccines [17], and virus-like particle (VLP) vaccines [18], [19].

CHIKV contains a positive-sense single stranded RNA genome with a 26S sub-genomic sequence that codes for a single structural polyprotein. This structural polyprotein is processed auto-catalytically and by host cell furin and signalase to yield the individual structural proteins that assemble into CHIKV virions [4]. Virions are 60–70 nm in diameter and consist of an icosahedral nucleocapsid composed of 240 copies of capsid protein (C) and a host cell derived envelope containing 240 embedded heterodimers of envelope glycoprotein 1 (E1), and envelope glycoprotein 2 (E2) [3], [20]. E1/E2 heterodimers are presented as trimeric spikes on the surface of mature virions and infected cells, and this E1/E2 complex contains conformational epitopes that give rise to neutralizing antibodies following natural infection or experimental vaccination [18], [21], [22]. After cell receptor-mediated endocytosis of an infectious CHIKV particle, endosomal acidification drives an irreversible conformational change in E1/E2 that exposes the E1 fusion peptide to mediate fusion with cellular membranes and viral entry into the cytoplasm [4], [23]. This conformational change can disrupt structural epitopes recognized by neutralizing antibodies, which may be important for the design and production of an effective CHIKV vaccine [21].

Transient transfection of HEK293 cells with a single expression vector carrying the cDNA sequence coding for the CHIKV strain 37997 structural polyprotein is sufficient to produce budded, enveloped CHIKV VLPs that contain an epitope in E1/E2 recognized by the conformation-sensitive neutralizing antibody m242 [21], [22]. Moreover, these recombinant CHIKV VLPs are immunogenic in non-human primates [18] and are currently being evaluated in a Phase I clinical trial as an investigative vaccine for human prophylactic use (ClinicalTrials.gov Identifier NCT01489358). Although transient gene expression (TGE) in mammalian cell lines using plasmid DNA expression vectors has advanced significantly in recent years as a recombinant protein production technology, it is still limited by industrial challenges related to increasing recombinant protein yield, manufacturing scale, and reproducibility [24]. With an estimated need for at least 6 million doses per year in predominantly developing countries [3], a scalable and reliable production process will be critical for developing a prophylactic CHIKV vaccine. In this work, the baculovirus expression vector system (BEVS) was investigated as an alternative to TGE for CHIKV VLP production.

BEVS utilizes an engineered *Autographa californica* multiple nucleopolyhedrovirus (AcMNPV) vector to produce recombinant protein products in lepidopteran insect cell lines such as *Sf*21, *Sf*9, or High Five. Long used for large-scale recombinant protein production for research use [25] and veterinary vaccines [26], [27], [28], BEVS has recently emerged as a scalable industrial production platform for human vaccines with the regulatory agency approvals of an influenza vaccine (Flublok, Protein Sciences Corporation) and a human papillomavirus (HPV) vaccine (Cervarix, GlaxoSmithKline). The system has been used successfully to produce recombinant VLPs for a wide variety of viruses, including the HPV L1 VLP in Cervarix [29]. Both enveloped and non-enveloped VLPs have been produced with BEVS, using a single baculovirus vector or co-infection with multiple vectors. Co-expression of multiple subunits from a single baculovirus vector with multiple expression cassettes has also been reported, along with expression of polyproteins which are processed post-translation into individual mature subunits [30], [31]. Recombinant expression of CHIKV E1 and E2 via BEVS has been described previously [17], and production of CHIKV strain S27 VLPs using a recombinant baculovirus vector was also recently reported [32]. Herein we describe the effect of culture pH on production of CHIKV strain 37997 VLPs from baculovirus infected insect cells, and describe a novel high-pH adapted insect cell line and elevated pH production process that significantly enhanced production of CHIKV VLPs suitable for the development of a CHIKV VLP vaccine candidate.

## Materials and Methods

### Ethics Statement

All animal studies were conducted in accordance with the 8^th^ Edition Guide for the Care and Use of Laboratory Animals [33], were designed to minimize animal suffering, and were approved by the Merck research site (West Point, Pennsylvania, USA) Institutional Animal Care and Use Committee (IACUC) after consultation with a laboratory animal veterinarian.

### Insect Cell Lines and Recombinant Baculoviruses

*Spodoptera frugiperda* cell lines *Sf*21 (Kemp Biotechnologies) and *Sf*9 (Invitrogen) were cultivated in suspension in serum-free Sf-900II (Gibco) growth media. Cells were maintained and expanded in vented Erlenmeyer shake flasks (Corning) at 27°C in a shaking incubator (Kuhner) set to 80 RPM and a 2″ shaking diameter. A cDNA fragment coding for the CHIKV strain 37997 structural polyprotein (Genbank accession #AY726732.1) was synthesized (DNA2.0) with flanking 5′ EcoRI and 3′ XbaI sites, and was then restriction cloned into pFastBac1 (Invitrogen) to produce pFastBac-CHIKV37997. Recombinant baculovirus DNA was generated by Tn7 transposition in DH10Bac *E. coli* using the Bac-to-Bac system (Invitrogen), and the resulting bacmid was transfected into *Sf*9 cells using Cellfectin-II (Invitrogen) to produce infectious recombinant baculovirus AcMNPV-CHIKV37997. Baculovirus infectious titers were determined using a Guava EasyCyte8HT flow cytometer (Millipore) and a gp64 immunofluorescence Baculovirus Titer Kit (Expression Systems LLC). Immunofluorescence results were converted to plaque forming units (pfu) using the baculovirus standard and analysis template supplied with the Baculovirus Titer Kit. GFP-expressing baculovirus (AcMNPV-GFP, AB Vector) or empty vector baculovirus (AcMNPV-NC, AB Vector) were utilized as negative controls for immunofluorescence and protein analysis methods. Cell counts and cell diameters were determined using a Vi-CELL XR and accompanying image analysis software (Beckman Coulter) using the pre-loaded *Sf*21 image analysis algorithm. Population doubling time (PDT) was calculated using time course Vi-CELL XR counts of cultures during exponential growth and standard cellular growth curve fit equations [34]. Statistical analysis of Vi-CELL XR results was performed using Minitab 16 software (Minitab).

### Mammalian Cell Line and Expression Vector

HEK293 cells (293-F, Invitrogen) were cultivated and transfected in suspension in serum-free FreeStyle 293 medium (Gibco). Cells were maintained and expanded in vented Erlenmeyer shake flasks (Corning) at 37°C and 8% CO_2_ in a shaking incubator (Kuhner) set to 125 RPM and a 2″ shaking diameter. A mammalian expression vector was constructed by restriction sub-cloning the EcoRI/XbaI fragment used to produce pFastBac-CHIKV37997 into a pV1JNS-based [35] plasmid under control of the hCMV promoter to create pV1JNS-CHIKV37997. This expression vector was transfected into HEK293 cells using 293fectin (Invitrogen) and the manufacturer-supplied protocol to produce positive control cells and culture supernatants containing CHIKV structural proteins and VLPs, respectively. Mock transfections with the pV1JNS vector (CHIKV37997 cassette omitted) were utilized as negative controls for immunofluorescence and protein analysis methods. Cell counts and cell diameters were determined using a Vi-CELL XR and accompanying image analysis software (Beckman Coulter) using the pre-loaded HEK293 image analysis algorithm.

### Baculovirus Infection of *Sf*21 in pH-modified Sf-900II

Serum-free Sf-900II medium (Gibco) was obtained at a pH of 6.3 and was adjusted to different target pH levels: 1 N HCl (Sigma-Aldrich) was used to reduce pH to 6.0, and 1 N NaOH (Sigma-Aldrich) was used to increase pH to 6.6–6.8. Growth medium pH was measured using a calibrated pH meter and probe (Fisher Scientific Accumet), and the pH-adjusted medium was sterile filtered through a 0.2 μm Durapore membrane (EMD Millipore). *Sf*21 cells were centrifuged at 200× g, spent Sf-900II media was fully aspirated, and the cells were re-suspended in pH 6.0–6.8 formulations of Sf-900II. Re-suspended *Sf*21 cultures (at 3×10^6^ viable cells/mL) were inoculated with AcMNPV-CHIKV37997 in Sf-900II media at an MOI of 1 pfu per viable cell. 150 mL cultures were inoculated in 500-mL vented Erlenmeyer shake flasks (Corning). Inoculated cultures were incubated at 27°C in a shaking incubator (Kuhner) set to 80 RPM and a 2″ shaking diameter. Cell suspension samples were removed 72 hours post-infection for immunofluorescence flow cytometry. Harvest samples were removed 96 hours post-infection, centrifuged to remove cells, and submitted to qELISA analysis. Statistical analysis was performed using Minitab 16 software (Minitab).

### Adaptation of *Sf*21 to Elevated Culture pH

Serum-free Sf-900II serum-free medium (Gibco) was diluted 1:1 with a custom N,N-Bis(2-hydroxyethyl)-2-aminoethanesulfonic acid (BES) buffered minimal insect supplement solution (BES-MISS) consisting of 50 mM BES, 124 mM Sucrose, 5 mM Glucose, 50 mM NaCl, 20 mM KCl, 3 mM CaCl_2_, 10 mM MgSO_4_, 0.1% w/v Pluronic F-68. All BES-MISS components were biotechnology grade and sourced from Sigma-Aldrich. The resulting Sf-900II-BES-MISS medium was adjusted to the target medium pH of 6.6–7.0 by addition of 1 N NaOH (Sigma-Aldrich), followed by sterilizing filtration via a Steri-Cup filter unit (EMD Millipore). *Sf*21 cells were centrifuged gently to completely exchange into pH 6.6 Sf-900II-BES-MISS medium, and then were allowed to recover until suspension cell growth began to approach the normal 20–24 hour PDT of a control *Sf*21 culture in standard Sf-900II medium. During recovery, the pH-adjusted Sf-900II-BES-MISS medium was refreshed every 2–5 days to maintain adequate nutrient levels and prevent acidification of the medium due to cellular metabolic activity. The medium pH was progressively increased using the same procedure over a period of 2 months until the PDT in pH 7.0 medium stabilized at 20–24 hours, and then a high pH adapted cell bank was established in Sf-900II+7.5% DMSO (Sigma-Aldrich) freezing medium. Cell bank vials were frozen using a Mr. Frosty controlled rate freezing box (Nalgene) and frozen vials were transferred to the vapor phase of a liquid nitrogen freezer for long-term cryopreservation. This adapted cell bank was named *Sf*Basic to distinguish it from the parental *Sf*21 cell line. After recovery from cryopreservation, *Sf*Basic cells were cultivated in suspension in serum-free, pH 7.0 Sf-900II-BES-MISS growth media. Cells were maintained and expanded in vented Erlenmeyer shake flasks (Corning) at 27°C in a shaking incubator (Kuhner) set to 80 RPM and a 2″ shaking diameter. Cell counts, cell diameters, and trypan blue exclusion cell viabilities were determined using a Vi-CELL XR and accompanying image analysis software (Beckman Coulter) using the pre-loaded Sf21 image analysis algorithm. Population doubling time (PDT) was calculated using time course Vi-CELL XR counts of cultures during exponential growth and standard growth curve fit equations [34]. Statistical analysis of Vi-CELL XR results was performed using Minitab 16 software (Minitab).

### CHIKV VLP Production in Shake Flasks (SF) at Elevated pH

Serum-free *Sf*21 and *Sf*Basic cultures at 3×10^6^ viable cells/mL were inoculated with AcMNPV-CHIKV37997 in Sf-900II media at an MOI of 0.1 pfu per viable cell. 35 mL cultures were inoculated in 125-mL vented Erlenmeyer shake flasks (Corning), and 300 mL cultures were inoculated in 2-L vented Erlenmeyer shake flasks (Corning). Inoculated cultures were incubated for 24 hours at 27°C in a shaking incubator (Kuhner) set to 80 RPM and a 2″ shaking diameter to initiate the infection. Infected cells were centrifuged at 200× g, the spent Sf-900II media was fully aspirated, and cells were resuspended in pH 7.4 Sf-900II-BES-MISS for VLP production. Culture pH was maintained between 7.0–7.4 by monitoring the pH of samples via calibrated benchtop pH meter and probe (Fisher Scientific Accumet) and aseptically adding sterile 1 N NaOH at a rate of 15 μL of 1 N NaOH/pH unit/mL of culture. Samples were removed at 72 and 96 hours post-infection, centrifuged to remove cells, and submitted for qELISA analysis. Data points without explicit time-point indications are 96 hour post-infection harvest samples. Statistical analysis was performed using Minitab 16 software (Minitab).

### CHIKV VLP Production in Stirred Tank Bioreactors (STBR) at Elevated pH

Serum-free *Sf*Basic cultures at 3×10^6^ viable cells/mL were inoculated with AcMNPV-CHIKV37997 in Sf-900II media (Gibco) at an MOI of 0.1 pfu per viable cell. 2 L cultures were inoculated in BIOSTAT 3-L jacketed glass bioreactors (Sartorius) and controlled using a BIOSTAT MD2 bioreactor control system (Sartorius). The culture was agitated with two 5.8 cm diameter pitched blade, low-shear impellers (Sartorius) at a constant 100 RPM, and temperature was maintained at 27°C using a PID loop to control the jacket water temperature. Aeration and gas exchange were accomplished using a ring sparger supplying 50 sccm of air and an overlay port supplying 200 sccm of air. Dissolved oxygen was controlled at 40% saturation (relative to culture media at equilibrium with ambient air) by a PID-controlled gas flow controller delivering pure oxygen at 40–100 sccm via the ring sparger. 24 hours after inoculation, the culture was centrifuged at 200× g and Sf-900II media was fully exchanged for pH 7.4 Sf-900II-BES-MISS media to promote VLP production. Culture pH was subsequently controlled at 7.2 by a PID-controlled peristaltic pump delivering sterile 1 N NaOH (Sigma-Aldrich) as required by the process. Samples were removed at 48, 72, and 96 hours post-infection, centrifuged to remove cells, and submitted for qELISA analysis.

### CHIKV VLP Purification from *Sf*Basic Culture

Culture broth from *Sf*Basic infected with AcMNPV-CHIKV37997 was harvested via centrifugation, and Halt protease inhibitor (Thermo-Pierce) was added to the supernatant. Supernatant was further clarified using 0.45 and 0.22 μm Durapore filters (EMD Millipore) and EDTA was added to 2 mM concentration. This filtered material was concentrated 15-fold and exchanged over 10 diavolumes into 150 mM NaCl, 20 mM HEPES, pH 8.0 buffer (Sigma-Aldrich) using a 500 kDa ultrafiltration filter (GE Healthcare). The ultrafiltration product was treated with Benzonase endonuclease (EMD Millipore) for 18 hours at 4°C, followed by filtration using 0.45 and 0.22 μm Durapore filters (EMD Millipore). Filtrate was loaded to a Sephacryl S-400 HR size exclusion column (GE Healthcare) with a 300 mM NaCl, 20 mM HEPES, pH 8.0 mobile phase. The eluate was filtered with a 0.2 μm Durapore filter (EMD Millipore) and exchanged into 20 mM HEPES, pH 8.0 buffer using a Sephadex G25 column (GE Healthcare). This product was then loaded to a Q-Sepharose XL anion exchange column (GE Healthcare) and eluted via a linear NaCl concentration gradient from 0 to 300 mM over 30 column volumes. The eluate was concentrated 10-fold and exchanged over 10 diavolumes into 11 mM potassium phosphate, 25 mM sodium citrate, 218 mM sucrose, pH 7.2 buffer using a 500 kDa ultrafiltration filter (GE Healthcare). The ultrafiltration product containing CHIKV VLPs was filtered through a 0.2 μm Durapore membrane (EMD Millipore) and stored at −70°C until needed for analysis. Chromatographic separations were performed using an AKTA Explorer chromatography system (GE Healthcare).

### Antibodies

Peptides corresponding to regions of CHIKV capsid, E1, and E2 proteins were synthesized and conjugated to Keyhole limpet hemocyanin (KLH, Covance). Anti-capsid antibody Ab3840 was raised against peptide AQIPVHMKSDASKFTHEKPEG, anti-E1 antibody Ab3845 was raised against peptide CHPPKDHIVNYPASHTTL, and anti-E2 antibody Ab3850 was raised against peptide CHAAVTNHKKWQYNSPLVPRN. All peptide-KLH conjugates were emulsified in Freund's Complete Adjuvant (FCA, Covance) for initial injections and emulsified in Freund's Incomplete Adjuvant (FIA, Covance) for all subsequent booster injections. Animals were injected subcutaneously (SC) with a 500 μg peptide initial dose in FCA at Day 0, and subsequently injected SC with 500 μg peptide doses in FIA at Day 21, 42, and 63. Intermediate bleeds containing approximately 20 mL of serum were removed at Day 52 and Day 73, and a final bleed containing approximately 50 mL of serum was removed at Day 77. Antibodies were isolated from serum samples using Protein G Sepharose Fast Flow resin (GE Healthcare) and eluted into a pH 7.4 phosphate buffered saline (PBS) solution. Hybridoma cell lines producing monoclonal antibodies m242 and m10-18 [21],[22] were supplied by the NIH Vaccine Research Center through a Cooperative Research and Development Agreement (CRADA). After standard hybridoma culture, antibodies were harvested from the cell culture supernatant using Protein G Sepharose Fast Flow resin (GE Healthcare) and eluted into a pH 7.4 phosphate buffered saline (PBS) solution.

### CHIKV VLP Standard

A purified CHIKV strain 37997 VLP preparation produced by a similar process to Phase I clinical trial VLP materials was obtained for use as a VLP standard from the NIH Vaccine Research Center (VRC) through a Cooperative Research and Development Agreement (CRADA). Briefly, VLPs were produced by HEK293 cells using polyethylenimine (Polysciences) mediated transient transfection of a plasmid DNA construct described previously [18]. HEK293 cells were transfected in FreeStyle 293 (Gibco) after adaptation to suspension, serum-free growth in EX-CELL 293 medium (SAFC). The cell culture supernatant was harvested via centrifugation and then clarified using a 0.45 μm PVDF filter (EMD Millipore). The clarified supernatant was concentrated 5-fold and diafiltered into a sucrose phosphate buffer (11 mM phosphate, 7.2% w/v Sucrose, pH 7.0) and then loaded to a Q Sepharose XL anion-exchange column (GE Healthcare). While bound to the resin, the VLPs were washed with the phosphate buffer and phosphate buffer supplemented with Benzonase endonuclease (EMD Millipore). VLPs were eluted with a citrate phosphate buffer (11 mM phosphate, 25 mM citrate, 7.2% w/v sucrose, pH 7.2) and diafiltered over 10 volumes against citrate phosphate buffer (11 mM phosphate, 25 mM citrate, 7.2% w/v sucrose, pH 7.2). The VLPs were then filter sterilized using 0.22 μm PVDF filters (EMD Millipore) and stored at −70°C until further use.

### SDS-PAGE and Western Blot

*Sf*21 cell lysates and culture supernatant samples were denatured for SDS-PAGE separation by mixing with Tris-Glycine reducing sample buffer (Invitrogen) containing SDS and DTT and heating for 10 minutes at 75°C. Denatured samples were loaded into a 4–20% Tris-Glycine pre-cast gel (Invitrogen) with MagicMark XP and Novex Sharp Pre-stained molecular weight markers (Invitrogen). Equivalent cell culture volumes or density gradient ultracentrifugation fraction volumes were loaded in each well to facilitate qualitative image-based comparisons. After electrophoresis, proteins were transferred to a nitrocellulose membrane using the iBlot transfer device and stack (Invitrogen). Membranes were blocked for 2 hours at room temperature using 5% nonfat dry milk (Bio-Rad) in Tris-buffered saline with Tween-20 (TBST, Santa Cruz Biotech), and then washed 3×5 minutes in TBST. Washed membranes were incubated with primary antibodies diluted in TBST for 2 hours at room temperature. Anti-capsid Ab3840, anti-E1 Ab3845, and anti-E2 Ab3850 were used at a 1∶430 dilution, and anti-Chikungunya 181/25 pAb (IBT Bioservices) was used at a 1∶1000 dilution. Membranes were washed 3×5 minutes in TBST and then treated for 2 hours with goat anti-rabbit IgG monoclonal antibody-alkaline phosphotase (AP) conjugate (Santa Cruz Biotech), diluted 1∶2000 in TBST. Membranes were washed 3×5 minutes in TBST, and then developed in NBT/BCIP 1-Step (Thermo-Pierce) for 5 minutes. The reaction was quenched by rinsing with distilled water, and developed blot membranes were scanned using an ImageScanner II imager with accompanying LabScan software (GE Healthcare). Dashed lines indicate different sections of the same gel. SDS-PAGE purity gels for purified VLP preparations were stained with Coomassie Blue, scanned using an ImageScanner II imager with accompanying LabScan software (GE Healthcare), and all protein bands were quantified using ImageQuant analysis software (GE Healthcare). SDS-PAGE purity is expressed as the sum of all CHIKV protein band intensities divided by the sum of all protein band intensities.

### CHIKV VLP Quantitative ELISA (qELISA)

A quantitative enzyme-linked immunosorbent assay (qELISA) was used to quantify assembled VLPs using neutralizing antibodies directed at spatially overlapping conformational epitopes on E2 when assembled in the E1/E2 complex. Total protein content for the HEK293-derived VLP standard was quantified using a BCA Protein Assay kit (Thermo-Pierce) and the manufacturer-supplied protocol. A BCA standard curve was prepared by independent dilution of 2 mg/mL albumin to 0.5–500 μg/mL. Standards and samples were plated on the plate (25 μL) and mixed with 200 μL of the BCA working reagent. The plate was shaken, incubated at 37 ± 5°C for 30 minutes, cooled to room temperature (RT), and read by a plate reader. SOFTmaxPRO software (Molecular Devices) was used to analyze the results, and a quadratic fit was used for the standard curve. 384-well microplates were coated at ambient temperature for 60 minutes with m242, an E2-specific neutralizing antibody. After washing and blocking, fifteen 1.67-fold serial dilutions of the VLP standard and test articles were generated, and 75 μL per well of each dilution was plated. After a 60 minute incubation and a plate wash step, 30 μL of 0.5 μg/mL biotinylated m10–18, another E2-specific neutralizing antibody, was added to the plate. The complex was washed, and signal was developed by the addition of streptavidin-alkaline phosphatase conjugate and the fluorogenic substrate, 4-methylumbelliferyl phosphate (4-MUP). A standard curve was generated by plotting fluorescence intensity (excitation 360 nm/emission 465 nm) as a function of the logarithm of analyte concentration. The resulting curve was fit with a four-parameter logistic equation, and unknown sample concentrations were determined by interpolation from the VLP standard curve. The limit of quantitation (LOQ) was determined to be 2 ng/mL relative to the VLP standard, and *Sf*21, *Sf*Basic, and HEK293 negative control samples (i.e. supernatants from cultures not expressing CHIKV proteins) were confirmed as less than LOQ.

### Immunofluorescence Flow Cytometry

Immunofluorescence surface staining with m242 was utilized as an indicator of the quantity of pre-fusion, conformationally correct E1/E2 complex displayed on the plasma membrane of cells. To prevent internalization of antibodies, all wash, block, and stain solutions were cold (2–8°C) and samples were kept on ice. AcMNPV-CHIKV37997 and AcMNPV-NC infected *Sf*21 cells were harvested after 3 days by gentle centrifugation and washed once with pH 7.2 PBS+1% Blocker BSA (Thermo). HEK293 cells transfected with pV1JNS-CHIKV37997 or mock transfected were harvested after 3 days and washed once with pH 7.2 PBS+1% Blocker BSA (Thermo). Washed cells were re-suspended in a preparation of m242 at 7 μg/mL in pH 7.2 PBS (1∶250 dilution) and incubated at 2–8°C for 2 hours. The cells were washed twice with pH 7.2 PBS+1% Blocker BSA, labeled with a goat anti-mouse IgG monoclonal antibody-AlexaFluor 488 conjugate (Molecular Probes), and incubated at 2–8°C for 2 hours. Labeled cells were washed twice with pH 7.2 PBS+1% Blocker BSA, and then analyzed immediately using a Guava EasyCyte8HT capillary flow cytometer (Millipore). AlexaFluor488 green fluorescence data was produced using GuavaSoft 2.2 software (Millipore), and statistical analysis was performed using Minitab 16 software (Minitab).

### Dynamic Light Scattering (DLS)

Purified VLP preparations derived from *Sf*Basic and HEK293 were loaded directly into a 40 μL low-volume quartz cuvette (Malvern) and analyzed in triplicate using a ZetaSizer Nano and accompanying software (Malvern Instruments). Standard protein material and water dispersant parameters were applied from the software package, and triplicate analyses were averaged for visualization of the size distribution. Size distribution data was exported to Minitab 16 software (Minitab) for calculation of mean particle diameter and 95% confidence intervals and for statistical hypothesis testing.

### Cell Cycle and Propidium Iodide Analysis

Un-infected *Sf*21 and *Sf*Basic cells were fixed and permeabilized for one hour at 2–8°C in 70% ethanol in PBS (Sigma-Aldrich). A Guava Cell Cycle Kit (Millipore) was used to stain the cells for flow cytometry analysis using a Guava EasyCyte8HT capillary flow cytometer (Millipore) and the manufacturer-supplied cell cycle procedure. Cell cycle data was analyzed using the standard Cell Cycle program from the GuavaSoft 2.2 software package (Millipore). Ethanol-fixed *Sf*21 and *Sf*Basic cells were also independently stained with propidium iodide (Molecular Probes) and imaged using a propidium iodide filter set and a fixed exposure time and magnification on an IX70 fluorescence microscope (Olympus) with SPOT 4.7 image capture software (SPOT Imaging Solutions).

### Density Gradient Ultracentrifugation

Sucrose density gradients spanning a calculated density range from 1.16–1.20 g/mL were constructed in Ultra-Clear centrifuge tubes (Beckman) by standard gradient methods. The sucrose gradient was generated in a 150 mM NaCl, 10 mM Tris, 1 mM EDTA, pH 8.0 buffer solution (Sigma-Aldrich). Culture supernatants from *Sf*Basic or HEK293 VLP production cultures were treated with 0.2 volumes of 5 M NaCl (Sigma-Aldrich) on ice for 10 minutes and then gently layered on top of the sucrose gradient solution. Loaded gradient tubes were centrifuged at 50,000× g for 4 hours in a SW41Ti rotor (Beckman) controlled at 16°C throughout centrifugation. Fractions were collected for Western blot analysis, and sucrose solution densities were confirmed using a refractometer (Thermo Scientific).

### VLP Electron Microscopy and Analysis

Electron microscopy was performed at NanoImaging Services (La Jolla, California, USA). Purified VLP samples were preserved in vitrified ice supported by holey carbon films on 400-mesh copper grids. Samples were prepared for imaging by applying a 3 μL drop of sample suspension to a cleaned grid, blotting with filter paper, and immediately proceeding with vitrification in liquid ethane. Grids were stored under liquid nitrogen until transfer to the electron microscope for imaging. Electron microscopy was performed using an FEI Tecnai T12 electron microscope, operating at 120 keV equipped with an FEI Eagle 4 k×4 k CCD camera. Vitreous ice grids were transferred into the electron microscope using a cryostage that maintains the grids at a temperature below −170°C. Images of each grid were acquired at multiple scales to assess the overall distribution of the specimen. After identifying potentially suitable target areas, pairs of high magnification images were acquired at nominal magnifications of 52,000× (0.21 nm/pixel) and 21,000× (0.50 nm/pixel). The images were acquired at a nominal underfocus of −4 μm (52,000×) and −5 μm (21,000×) and electron doses of 10–25 e^−^/Å^2^.

Individual particles in the 21,000× magnification images were selected using automated picking protocols [36]. A reference-free alignment strategy based on the XMIPP processing package [37] was then applied. Algorithms in this package aligned the selected particles and sorted them into self-similar groups of classes. The XMIPP package uses the Kernel Probability Density Estimator Self-Organizing Map (KerDenSOM) classification method [38], which maps a set of high dimensional input vectors into a regular two-dimensional grid so that the proximity of the units in the map reflects the similarity of the input data. *Sf*Basic-derived particles and HEK293-derived VLP standard particles were counted and evaluated, and 2D class averaged VLP images were produced from 148 and 199 high quality particle images for *Sf*Basic-derived and HEK293-derived VLPs, respectively. Image-based fractional counting of putative CHIKV particles (round, 50–70 μm in diameter) and baculovirus particles (rod-like, 300–400 nm in length) was also performed for a set of images from the *Sf*Basic-derived sample, including 422 total particles.

### Thin-Section TEM of Cells

Electron microscopy was performed at NanoImaging Services (La Jolla, California, USA). AcMNPV-CHIKV37997 infected *Sf*21 cells in pH 6.3 Sf-900II (Gibco) and pV1JNS-CHIKV37997 transfected HEK293 cells were fixed overnight at 4°C using 2.5% glutaraldehyde in 0.1 M Sodium cacodylate buffer with 1 mM CaCl_2_, pH 7.3 (NanoImaging Services). Samples were prepared using standard embedding and thin sectioning procedures and a continuous carbon grid method. Grids were nitrocellulose supported 400-mesh and slotted copper. Samples were stained with 2% uranyl acetate for imaging. Electron microscopy was performed using an FEI Tecnai T12 electron microscope, operating at 120 keV equipped with an FEI Eagle 4 K×4 K CCD camera. Negative stain grids were transferred into the electron microscope using a room temperature stage. Images of each grid were acquired at multiple scales to assess the overall distribution of the specimen. After identifying potentially suitable target areas, pairs of high magnification images were acquired at nominal magnifications of 52,000× (0.21 nm/pixel), 21,000× (0.50 nm/pixel), and 15,000× (0.71 nm/pixel). The images were acquired at a nominal underfocus of −4 μm (52,000×), −5 μm (21,000×) and −10 μm (15,000×) and electron doses of 2–30 e^−^/Å^2^. A high magnification tilt series was acquired at a nominal magnification of 21,000× (0.50 nm/pixel), nominal underfocus of −5 μm, and electron doses of 2–4 e^−^/Å^2^.

### Animals and Vaccination

Hartley guinea pigs were obtained from Charles River Laboratories. Purified CHIKV VLPs derived from infection of *Sf*Basic with AcMNPV-CHIKV37997 and VLP standard derived from transient transfection of HEK293 cells were adjuvanted onto Adju-Phos aluminum based adjuvant (Brenntag Biosector). Guinea pigs (4 animals per group) were vaccinated intramuscularly with doses of 0.01, 0.1, 1, or 10 μg of CHIKV VLPs. Animals were vaccinated at Day 0 and Day 14, and serum was sampled on Day 14 (prior to dosing) and on Day 21 (at study completion). A pre-vaccination serum sample was taken prior to the first vaccination for the purpose of establishing the serum IgG ELISA background.

### Serum IgG ELISA

Anti-CHIKV IgG titers were determined by immobilizing purified CHIKV VLPs onto a plate and determining the antigen binding endpoint IgG concentration of serum samples using a standard enzyme-linked immunosorbent assay (ELISA) format in duplicate [39]. Briefly, Maxisorp 96F plates (Nunc) were coated with 0.2 mg/mL of CHIKV VLP standard in PBS overnight and blocked with 1% BSA in PBS with 0.05% Tween 20. 100 μL of serial dilutions of guinea pig serum samples were added to the wells, incubated for 1 hour, and washed. Bound guinea pig IgG was detected using a goat anti-guinea pig IgG horesradish peroxidase conjugate (Jackson Laboratories). Signal was developed from bound peroxidase using the chromogenic substrate 3,3′,5,5′-tetramethylbenzidine (Thermo-Pierce) and sulfuric acid to quench. Plates were read at 450 nm using a spectrophotometer (Beckman Coulter), and the serum titer was determined by taking the reciprocal of the highest dilution factor which produced a signal at least 3-fold greater than background signal. Graphs and statistics were generated with the GraphPad Prism 5 software package (GraphPad Software). Geomean antibody titers (N = 4 animals per group) are reported graphically with each animal represented by a data point [40], [41], and the nonparametric Kruskal-Wallis test was applied with Dunn's post-test for pairwise comparisons between dose-matched groups vaccinated with VLPs derived from HEK293 or *Sf*Basic. Indications of background signal represent the geomean titer of pre-vaccination serum samples for all animals. A single statistical outlier (Grubbs' test [42] in GraphPad Prism 5, p<0.01) was removed from the data set (HEK293 derived VLP, 0.01 μg dose level), but is still indicated by a data point in plots.

### Serum Neutralization Assay

Guinea pig sera were analyzed in duplicate using a 100% neutralization titration (NT100) with CHIKV strain 181/25 [11]. One day prior to CHIKV infection, Vero cells (American Type Culture Collection) were plated at 15,000 cells per well in a 96 well plate (Nunc). Neutralization titers were determined by mixing serial dilutions of guinea pig sera with 350 PFU of CHIKV 181/25 and incubating for 1 hour at 37°C. After the incubation, samples were added to Vero cell monolayers and incubated for 3 days. Vero cell monolayers were subsequently fixed and stained with 0.05% crystal violet, 20% methanol (Sigma-Aldrich). Neutralization titers were determined by taking the reciprocal of the last dilution where the Vero cell monolayer remained fully intact. Graphs and statistics were generated with the GraphPad Prism 5 software package (GraphPad Software). Geomean titers (N = 4 animals per group) are reported graphically with each animal represented by a data point [40], [41], and the nonparametric Kruskal-Wallis test was applied with Dunn's post-test for pairwise comparisons between dose-matched groups vaccinated with VLPs derived from HEK293 or *Sf*Basic.

## Results and Discussion

### Expression and Processing of Recombinant CHIKV Structural Proteins in *Sf*21

**Figure 1:**
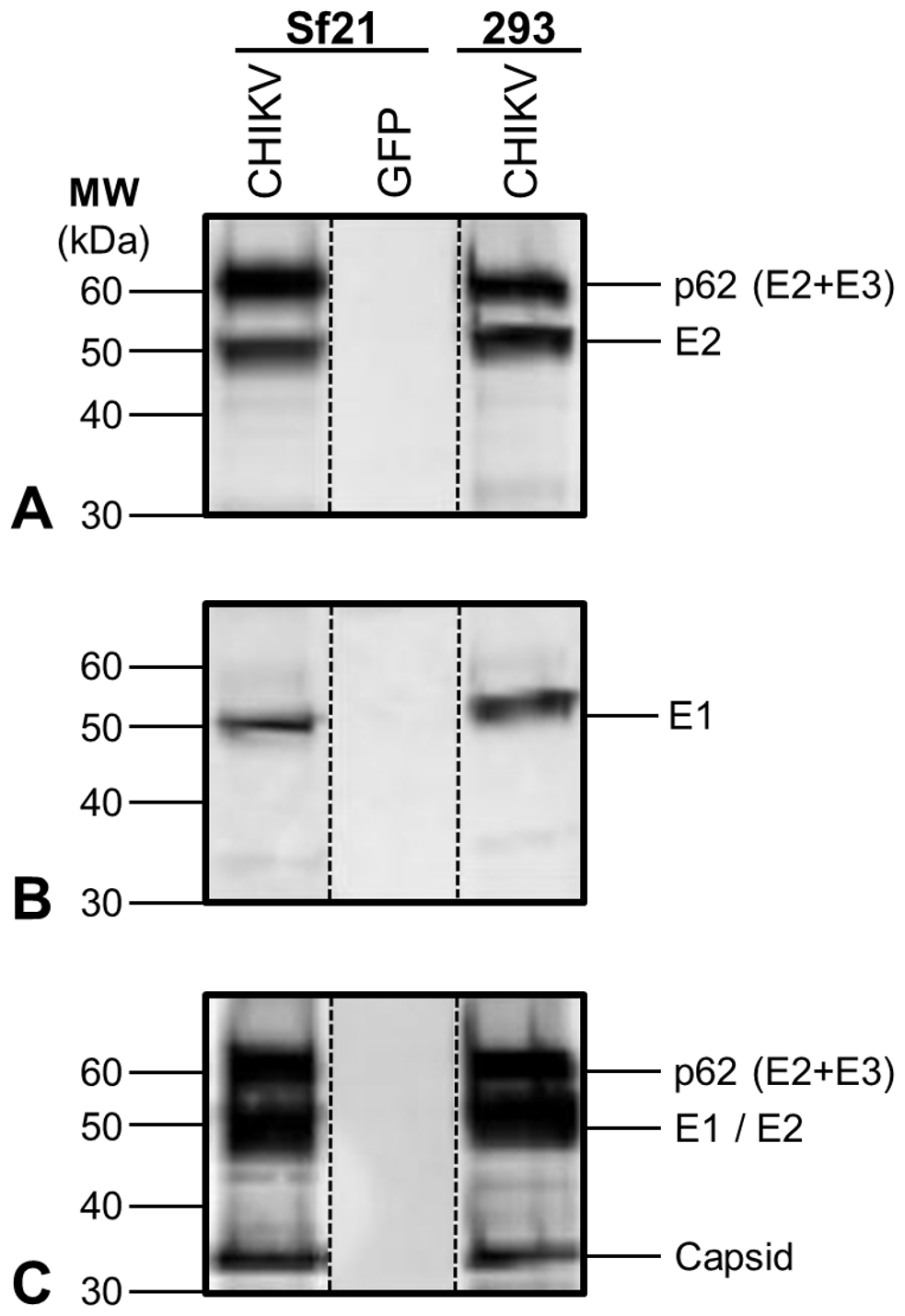
CHIKV structural polyprotein expression and processing in AcMNPV-CHIKV37997 infected *Sf*21 cells and pV1JNS-CHIKV37997 transfected HEK293 cells. Cell lysate Western blots depicting (**A**) E2 expression and processing, detected by an E2 peptide-specific antibody. (**B**) E1 expression, detected by an E1 peptide-specific antibody. (**C**) E1/E2 (co-migrating) and capsid expression and processing, detected by an anti-CHIKV polyclonal antibody. AcMNPV-GFP infected *Sf*21 lysate was included as a negative control for insect cells and baculovirus vector.

A Phase I clinical trial is underway for evaluation of a CHIKV VLP vaccine produced by transient transfection of HEK293 cells. However, recombinant production of CHIKV VLPs by plasmid-based transient gene expression (TGE) in mammalian cells presents some technical challenges for industrial scale vaccine manufacturing [24]. To develop an alternative production platform, the cDNA sequence coding for CHIKV strain 37997 structural polyprotein was inserted into a recombinant baculovirus vector to generate AcMPNV-CHIKV37997. As shown in Figure 1, the strain 37997 structural polyprotein was expressed and processed into individual structural proteins intracellularly when *Sf*21 cells were infected in standard Sf-900II medium with AcMNPV-CHIKV37997. CHIKV E2 was correctly processed at the furin recognition site to cleave E3 from E2, but the presence of a band consistent with p62 (E3/E2) suggests incomplete cleavage. The same p62 band was also detected in the HEK293 cell positive control lysate on the same gel, indicating that incomplete processing by furin in *Sf*21 cells is unlikely to preclude the production of CHIKV VLPs. CHIKV E1 was produced by infected *Sf*21 cells, and CHIKV capsid protein was also expressed and auto-catalytically cleaved from E3 as expected.

A quantitative ELISA (qELISA) was used to determine the concentration of VLPs using anti-E2 neutralizing antibodies m242 and m10–18, which bind spatially overlapping conformational epitopes presented by the pre-fusion E1/E2 complex. Binding of antibody m242 has been reported to prevent E1/E2 conformational change [21]. The binding sites for these antibodies have also been defined in a 5.3 Å resolution cryo-electron microscopy (cryoEM) map of CHIKV VLPs [22], confirming their specificity for detection of epitopes presented on VLPs.

**Figure 2:**
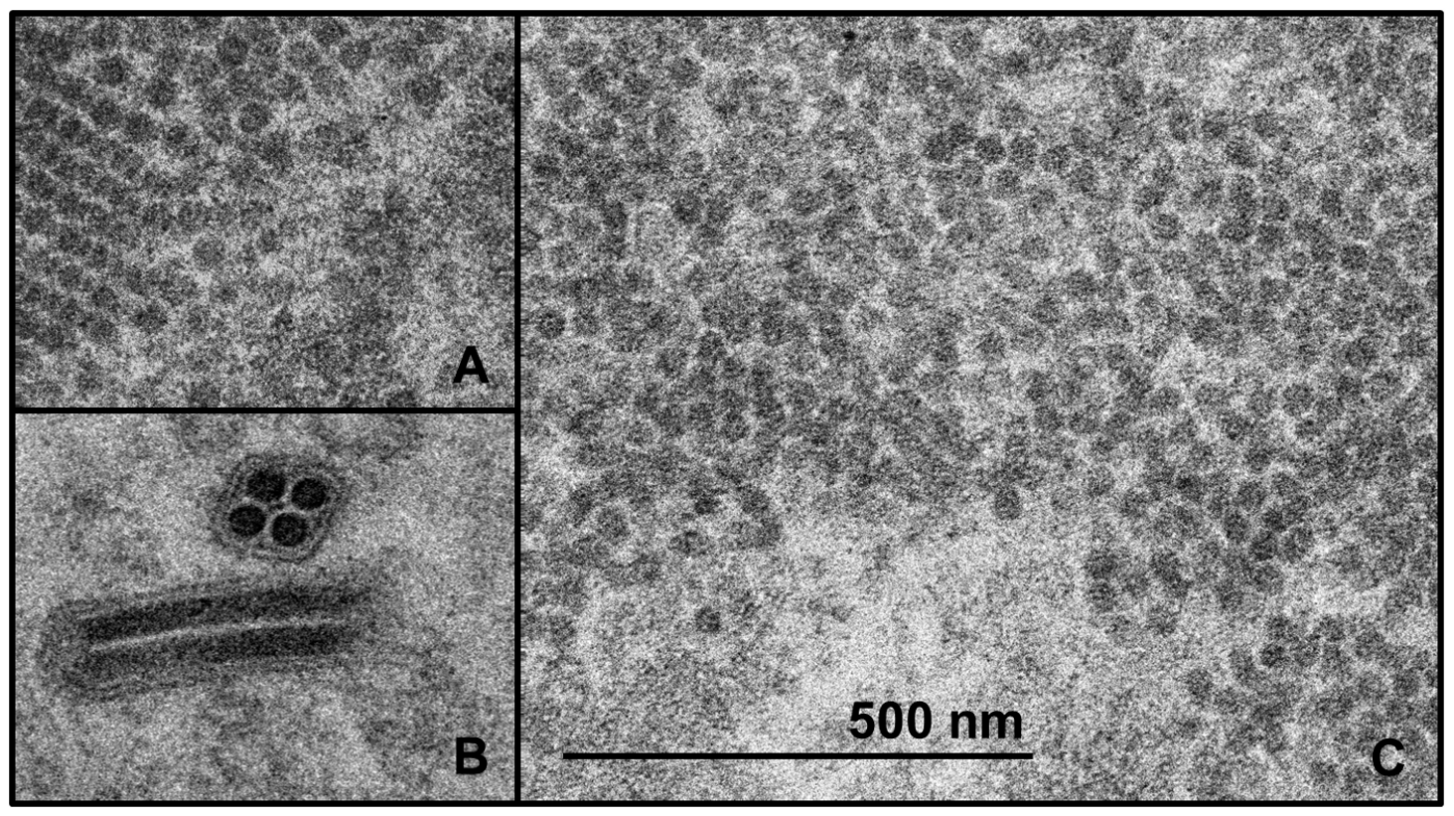
Transmission electron microscopy (TEM) images of thin-sections of AcMNPV-CHIKV37997 infected *Sf*21 cells and pV1JNS-CHIKV37997 transfected HEK293 cells. (**A**) Putative CHIKV capsids formed in the cytoplasm of HEK293 cells. Capsid diameters are approximately 30–35 nm. (**B**) Baculovirus showing hallmark multiple nucleocapsids per envelope, with very electron dense nucleocapsids. Nucleocapsid diameter is approximately 40 nm. (**C**) Putative CHIKV capsids formed in the cytoplasm of *Sf*21 cells. Capsid diameters are approximately 30–35 nm. Scale bar is equivalent for all images and represents 500 nm.

Despite the presence of processed E1, E2, and capsid protein in *Sf*21 cell lysates, the corresponding *Sf*21 culture supernatants produced no detectable signal in the sensitive, VLP-indicating qELISA (LOQ = 2 ng/mL). However, thin-section TEM images of the cytoplasm of AcMNPV-CHIKV37997 infected *Sf*21 cells (Figure 2) revealed the formation of large clusters of approximately 30–35 nm diameter particles that were less electron dense than baculovirus nucleocapsids. Similar particles and clustered arrays were observed to be prevalent in transfected HEK293 cells that produced budded CHIKV VLPs, but were not observed in surveys of negative control images of *Sf*21. These putative recombinant CHIKV capsids were consistent with previous descriptions of CHIKV capsids [43], suggesting that capsid formation and organization in the cytoplasm was likely not responsible for preventing VLP budding into the supernatant.

A repeat infection performed in cholesterol-supplemented growth media produced similar results to the standard infection process (data not shown), despite the cholesterol dependence for budding of the related SINV and SFV [44], [45], [23]. Acylation of E1 and E2 is also involved in alphavirus budding [46], [47], but was not investigated in this work due to the documented ability of insect cells to palmitoylate the glycoproteins of AcMNPV, Marburg virus, and SFV [48],[49], [50]. Taken together, these observations suggested that *Sf*21 cellular or culture conditions were influencing the conformation and stability of the E1/E2 complex thought to be involved in budding [21].

### Effect of Culture pH on CHIKV VLP Production from *Sf*21

Increasing cell culture medium pH over the range of 6.5 to 8.0 has been previously demonstrated to significantly increase the budding of Semliki Forest virus (SFV) from Baby Hamster Kidney (BHK) cells [51]. A similar increase in production of Sindbis virus (SINV) and Venezuelan equine encephalitis virus (VEEV) over the pH range 6.8–7.9 has been reported from BHK cells and chick embryo fibroblasts (CEF) [52]. Changes in pH up to 7.9 also yielded increases in CHIKV VLP production from transfected HEK293 cells, potentially due to the stabilization and retention of the correct pre-fusion conformation in the E1/E2 glycoprotein complex [21]. *Sf*21 and *Sf*9 for use in human biopharmaceutical or vaccine production are typically cultured in suspension in various published or commercially available serum-free growth medium formulations which utilize a phosphate buffer system to maintain a slightly acidic culture pH in the optimal range of 6.0–6.4 [53], [54]. A broader pH requirement of 6.0–6.8 for various insect cell lines has also been previously described, with deleterious effects on cell growth and viability reported upon deviations outside of this range [55].

The intracellular pH of *Sf*21 cells remains at or near 7.0 in response to extracellular pH variation from 6.2 to 6.8 and is not affected by baculovirus infection [56]. However, based on the slightly acidic nature of insect cell growth media and previously reported sensitivity of the CHIKV E1/E2 complex and alphavirus budding to extracellular pH [21], [51], [52], the effects of elevated culture pH on cell surface display of CHIKV glycoproteins and on VLP production were examined. While strain 37997 VLP yield was not affected significantly by modulation of pH in the range of 7.0–7.9 when expressed in HEK293 cells [21], the much lower pH 6.0–6.4 range of insect cell growth medium allowed for the possibility that increasing pH toward or into the typical mammalian cell culture pH range could be beneficial in this expression system.

**Figure 3:**
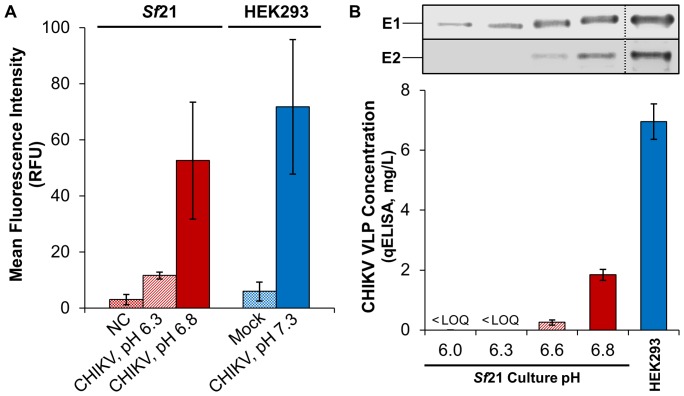
Effect of elevated culture pH on cell-surface localization of CHIKV glycoproteins and CHIKV VLP yield. (**A**) Mean fluorescence intensity (MFI) from surface staining of AcMNPV-CHIKV37997 infected *Sf*21 and pV1JNS-CHIKV37997 transfected HEK293 cells with neutralizing antibody m242. AcMNPV-NC and a mock transfection were included as negative controls for *Sf*21 and HEK293, respectively. Error bars represent 95% confidence intervals (N = 3 independent infections/transfections). (**B**) E1/E2 Western blot and VLP concentration for supernatants from infected *Sf*21 and transfected HEK293. Error bars represent 95% confidence intervals (N = 4 assay replicates). <LOQ indicates a result less than the qELISA limit of quantitation, or <2 ng/mL.

When *Sf*21 was infected with AcMNPV-CHIKV37997 in unmodified culture media at pH 6.3, mean fluorescence intensity (MFI) from cell surface immunofluorescence staining with neutralizing antibody m242 was observed to be only 4-fold greater than the negative control (NC) background and substantially lower than the HEK293 positive control transfection (Figure 3). No visible E2 band was detected by Western blot of *Sf*21 culture supernatants at pH 6.0–6.3, but a faint E1 antibody-reactive band was detected. However, no quantifiable qELISA signal was observed in this pH range, suggesting that the weak E1 signal by Western blot could have resulted from the release of low levels of intracellular E1 due to the lytic baculovirus infection process.

When culture pH was increased from 6.3 to 6.8, MFI from m242 surface staining of *Sf*21 increased to 22-fold over the NC background and became more similar in magnitude to transfected HEK293. Upon increase from pH 6.3 to pH 6.6 and 6.8, E1 and E2 bands were detected in increasing intensity by Western blot of *Sf*21 supernatants, and qELISA indicated the production of an increasing concentration of CHIKV VLPs. Despite these consistent increases with elevated culture pH, the E1 and E2 Western blot band intensities and qELISA signal for *Sf*21 at pH 6.8 still did not reach the same protein or VLP production levels as the HEK293 positive control. The increase in conformational E1/E2 complex detected on *Sf*21 cell surfaces and increase in budded VLP production as functions of culture pH suggest that stabilization of the E1/E2 complex may contribute to budding of CHIKV strain 37997 VLPs from baculovirus infected *Sf*21 cells.

A recent investigation of CHIKV VLPs produced via baculovirus infection of insect cells did not report this elevated pH requirement for production of VLPs [32], [19]. However, the experiments of Metz et al were conducted using a different *Sf*21 source (Invitrogen), baculovirus vector (Δp10Δcc), culture medium formulation (Grace's), cell culture format (adherent monolayer), and Chikungunya polyprotein sequence (S27). While VLPs were produced in serum-free conditions, growth of the cells in the presence of undefined fetal bovine serum (5% FBS) may also complicate comparisons to the entirely serum-free propagation and production methods used in this work. Analytical methods also diverge significantly from those described herein, particularly in the antibodies and procedures used to quantify VLPs. The requirement for elevated pH observed in our work with strain 37997 VLPs cannot currently be generalized to other strains of CHIKV and insect cell production conditions, but due to the accumulated reports of a pH effect on CHIKV particle production in BHK, CEF, HEK293, and *Sf*21, pH-based enhancement of VLP production for strains other than 37997 (including S27) and for differing expression conditions may warrant further investigation.

### Adaptation of *Sf*21 to Elevated Culture pH

CHIKV VLPs were produced from infected Sf21 cells and detected by qELISA at pH 6.6–6.8, but this culture pH range is outside the reported optimum for Sf9 and Sf21 cell lines and is approaching the reported limit of normal physiology for cultured insect cells [55]. Due to the heterogeneous nature of the *Sf*21 cell line [57], [58], it was hypothesized that applying pH stress gradually over many passages in a suitable growth medium could allow cells to adapt or be selected to produce CHIKV VLPs more effectively in an elevated culture pH range.

Adjustment of Sf-900II medium to pH 6.6–6.8 by direct addition of hydroxide and subsequent sterile filtration was sufficient to demonstrate increasing CHIKV VLP production with increasing culture pH in initial experiments, but visible and significant precipitation of culture medium components was observed when Sf-900II was adjusted to higher pH levels. This pH-induced precipitation resulted in medium instability during storage and to decreased *Sf*21 cell growth and culture viability, thus necessitating a reformulation of the growth medium to extend the accessible operating culture pH range. The potential for precipitation of calcium phosphate species and other divalent cation phosphates from phosphate-buffered growth medium during pH adjustment is well known in the cell culture field. To address this, a modified Sf-900II growth medium with reduced phosphate concentration (Sf-900II-BES-MISS) was developed for this work using N,N-bis(2-hydroxyethyl)-2-aminoethanesulfonic acid (BES) as an alternative buffer. In addition to Sf-900II medium (Gibco), serum-free BaculoGold Max-XP (BD Biosciences) and ESF-921 (Expression Systems) media were also shown to support adequate cell growth at elevated pH when modified to reduce phosphate concentration in a similar manner to Sf-900II-BES-MISS. Several other Good's buffers [59], including MOPS, HEPES, TES, glycylglycine, and tricine, were also shown to be non-toxic and effective for replacing buffering capacity at concentrations up to 30–40 mM in reduced phosphate media (data not shown). Customized, supplemented Sf-900II-BES-MISS growth medium supported adjustment of culture pH to pH 7.0–7.4 with minimal precipitation and enabled the adaptation of *Sf*21 to a more elevated pH range than standard Sf-900II medium.

Over a period of six weeks of continuous and increasing pH stress spanning from pH 6.4 to pH 7.0 (in 0.2 unit steps), a stable high pH adapted insect cell line variant was derived, cryopreserved, and re-named *Sf*Basic to distinguish it from the parental *Sf*21. A slight decline in culture viability and total cells was observed by trypan blue exclusion upon initial exposure of *Sf*21 to culture pH of 6.6, but a significant proportion (89%) of cells remained viable. Culture viability recovered to 96–98% during adaptation, and has since stabilized in the same range for routine cultivation. This response suggests that *Sf*Basic resulted from both immediate stress-based selection and longer term stress-induced adaptation or increased tolerance over time.

**Figure 4:**
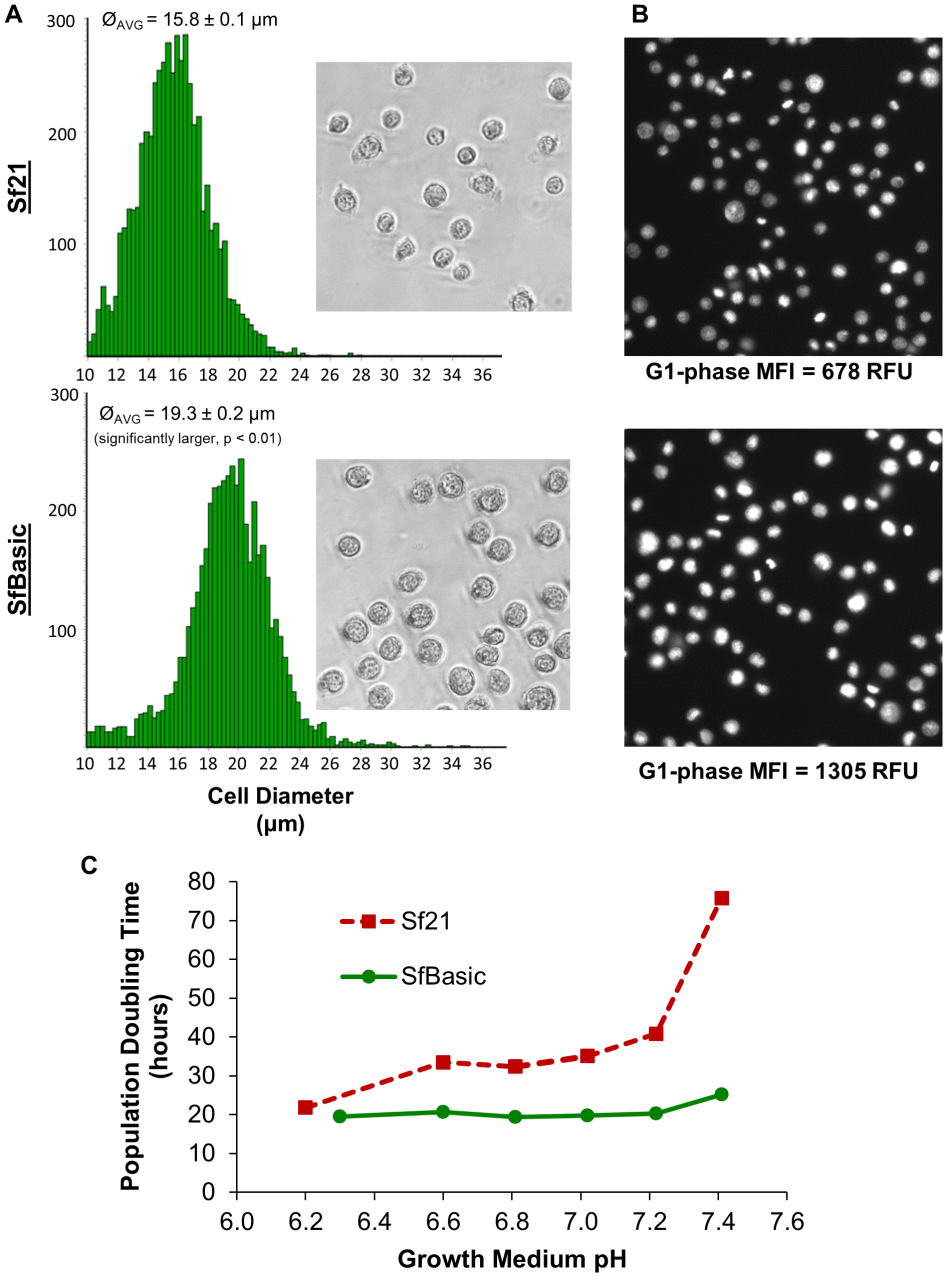
Characterization of the *Sf*Basic cell line and comparison to the parental *Sf*21 cell line. (**A**) Cell diameter (Ø) distribution histogram with a representative bright field image. (**B**) Fluorescence microscopy images of propidium iodide stained cells. Mean fluorescence intensity (MFI) of the G1-phase cell population from flow cytometry cell cycle analysis of the same sample is indicated below the corresponding image. (**C**) Cell line population doubling time (PDT) as a function of growth medium pH. The growth medium was Sf-900II-BES-MISS, adjusted to various pH levels by titration with 1 N NaOH.

As shown in Figure 4, the average cell diameter for *Sf*Basic is 3.5 μm larger than the parental *Sf*21 (19.3±0.2 μm vs. 15.8±0.1 μm), corresponding to an 82% increase in cellular volume. This difference in the average cell diameter of the pH-adapted and parental cell populations was established by Vi-CELL XR microscopy image analysis and is statistically significant (Two-Sample t-Test of 12 independent samples each, p<0.01). Propidium iodide (PI) stained *Sf*Basic cells also produced qualitatively higher PI signal intensities in traditional fluorescence microscopy images acquired at a fixed exposure time. When subjected to flow cytometry-based cell cycle analysis, *Sf*Basic cells in G1-phase gate yielded a 1.9-fold increase in mean fluorescence intensity over the parental *Sf*21. This shift in cell size distribution and increase in nucleic acid content to approximately 2N is consistent with previous reports of adaptation of *Sf*9 and *Sf*21 to elevated temperatures [60], [61], but to our knowledge, a pH-stress selection and adaptation strategy to derive a favorably modified insect cell population has not been published prior to this work.

*Sf*Basic retains the ability to grow normally under standard insect cell culture pH conditions, but also doubles at a consistent growth rate up to pH 7.2, after which the PDT increases slightly. The PDT for *Sf*21 first increases at pH 6.6 and then increases significantly from pH 7.0–7.4, demonstrating the previously reported sensitivity of insect cell lines to elevated pH [55]. The broadening of the normal growth range of *Sf*Basic as a function of pH suggests that this cell population may be the result of an overall increase in tolerance to pH, as opposed to a true adaptation and shift to a new optimum. The average cell diameter, culture viability, and growth rate of *Sf*Basic have currently been demonstrated to be stable for 30 passages (approximately 90 population doublings) after adaptation, and no observation of phenotypic instability has yet been observed during continuous passage.

### CHIKV VLP Production from *Sf*Basic pH-Adapted Cell Line

**Figure 5:**
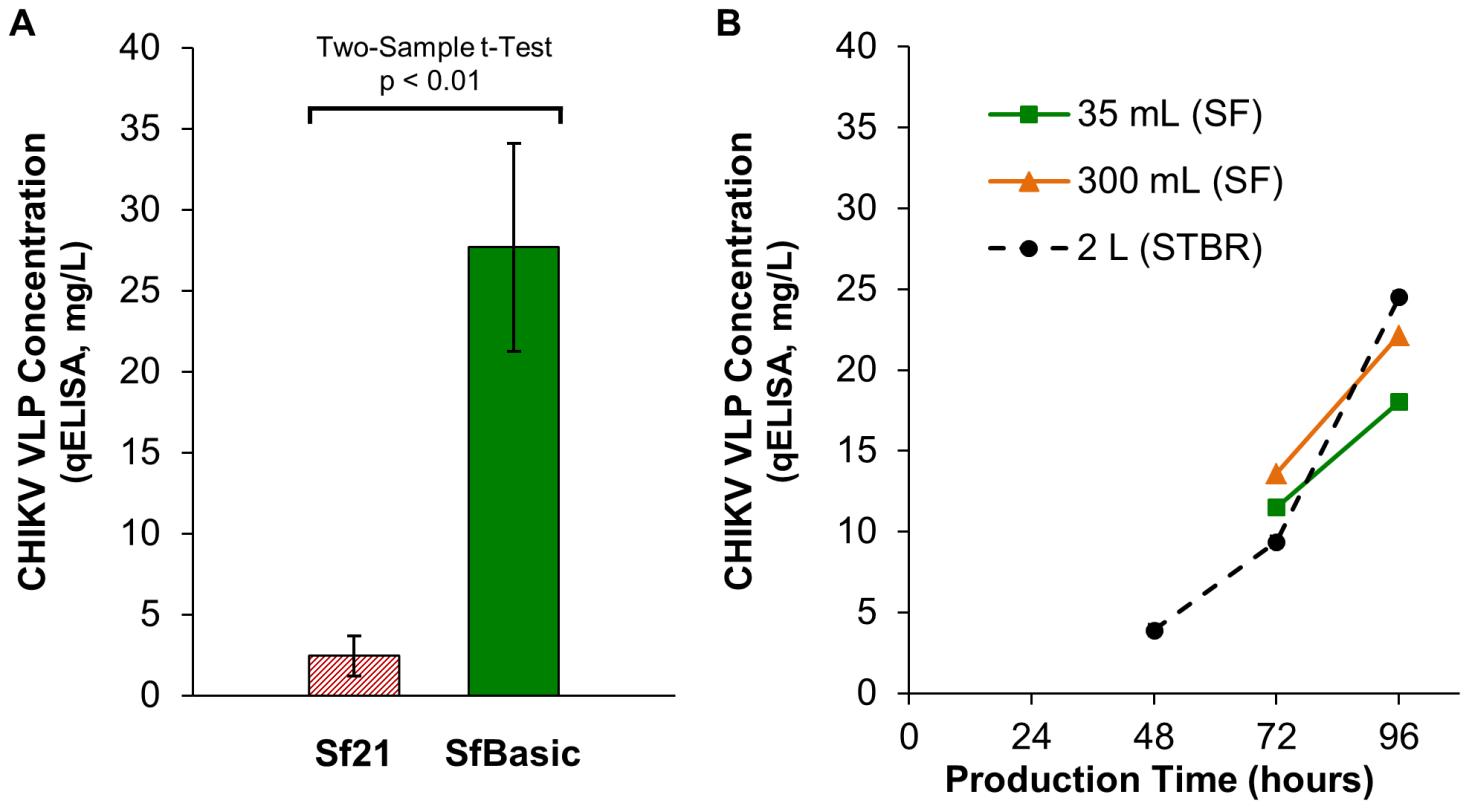
Volumetric CHIKV VLP yield enhancement resulting from adaptation of *Sf*21 to elevated culture pH. (**A**) CHIKV VLP concentration of culture supernatants from *Sf*21 and *Sf*Basic small-scale shake flask cultures. Error bars represent 95% confidence intervals (N = 10 independent infections). (**B**) CHIKV VLP concentration of culture supernatants from scale-up of *Sf*Basic from shake flasks (SF) into a PID controlled 3-L stirred tank bioreactor (STBR).

*Sf*Basic was adapted to grow normally at elevated culture pH, but robust cell growth under particular culture conditions is not a validated surrogate for baculovirus-mediated production of recombinant products. To determine the extent to which pH adaptation contributes to CHIKV VLP productivity, *Sf*21 and *Sf*Basic were subjected to infection with AcMNPV-CHIKV37997 in a modified high pH infection and production process. As shown in Figure 5, *Sf*Basic infection produced an 11-fold, statistically significant increase in volumetric CHIKV VLP yield relative to the parental *Sf*21 (Two-Sample t-Test, p<0.01). Very similar CHIKV VLP productivities were also achieved when this production process was scaled up from small-scale shake flask infections into larger shake flasks and stirred tank bioreactors. The 28 mg/L volumetric productivity of SfBasic was not significantly different from the 31 mg/L productivity of HEK293 (Two-Sample t-Test, p = 0.50). The specific yields of budded VLPs from SfBasic and HEK293 cells were also similar; SfBasic produced 9 μg of VLP per million infected cells and HEK293 produced 8 μg of VLP per million transfected cells (Two-Sample t-Test, p = 0.43).

**Figure 6:**
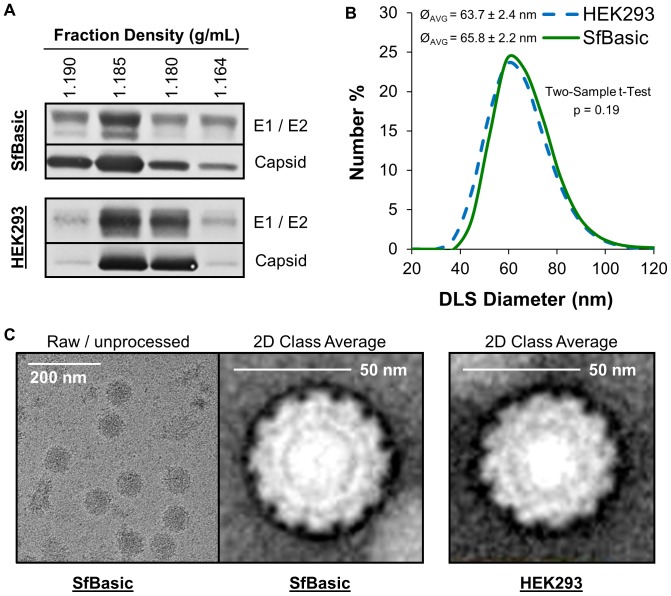
Biophysical characterization of CHIKV VLPs derived from SfBasic cells and comparison to a VLP standard derived from HEK293 cells. (**A**) Western blot of density gradient ultracentrifugation fractions containing CHIKV VLPs, using E1, E2, and capsid peptide-specific antibodies. (**B**) Dynamic light scattering (DLS) distribution of purified CHIKV VLP diameters (Ø). (**C**) Raw/unprocessed and 2D class average transmission electron microscopy images of purified CHIKV VLPs.

Scale-up of the *Sf*Basic suspension infection process into bioreactors supported chromatographic purification of large quantities of CHIKV VLPs with purification yields of 15–20%. Purified VLPs from *Sf*Basic had similar buoyant density to the HEK293-derived VLP standard when subjected to sucrose density gradient ultracentrifugation and Western blot detection (Figure 6). The 65.8±2.2 nm mean diameter of *Sf*Basic-derived VLPs determined by dynamic light scattering (DLS) was not significantly different from the 63.7±2.4 nm mean diameter of the VLP standard (Two-Sample t-Test, p = 0.19). 2D class averaging of electron micrograph images revealed that VLPs produced by *Sf*Basic demonstrate slight differences in electron density compared to the VLP standard, but that the overall VLP size, icosahedral symmetry, and structure are very similar. After chromatographic purification from *Sf*Basic culture, fractional counting of 422 particles in the electron microscopy images yielded a 97% CHIKV VLP fraction with 3% residual baculovirus by particle count. The SDS-PAGE protein purity for the final *Sf*Basic-derived VLP preparation was 83% and was within assay variability of the purity of the HEK293-derived VLP standard lot used for comparison throughout this work.

BEVS and the *Sf*Basic cell line have many desirable attributes for production of a CHIKV VLP vaccine, but there are challenges to overcome in the development of a purification process for human vaccine manufacture. Virus clearance and inactivation of recombinant baculovirus and model viruses can be especially difficult for enveloped VLP vaccines due to VLP sensitivity to chemical treatment and structural similarity to the enveloped baculovirus vector and some model viruses. Detergent treatment and low pH treatment are two common methods of virus inactivation in bioprocesses, but both of these operations are likely to disrupt the structure of the CHIKV VLP. Viral vector bioprocesses often employ density gradient ultracentrifugation fractionation, but CHIKV and AcMNPV have similar buoyant densities and would likely be difficult to separate consistently in a scalable, economically feasible ultracentrifugation process [18], [62], [63]. Chromatographic separation methods such as ion exchange chromatography (IEX) and size exclusion chromatography (SEC) were applied in this work and have potential for further optimization and scale-up, but the inherent charge and size similarity between the CHIKV VLP and other viruses also pose challenges for these methods. Encapsidation of host cell DNA and RNA has also been documented for VLPs produced by BEVS [64], [65], resulting in the potential for undesirable delivery of host cell nucleic acids into vaccine recipients. Aside from the removal of recombinant baculovirus vector, all of these bioprocess challenges also apply to the production of enveloped VLPs from mammalian cell lines. If adequate baculovirus clearance can be demonstrated, the use of an insect cell line as host for enveloped VLP production may even be advantageous relative to mammalian cell lines due to the decreased likelihood of contamination of the VLP drug product with nucleic acids or adventitious viruses that could be active in human vaccinees.

### Immunogenicity of CHIKV VLPs Derived from *Sf*Basic

The recombinant expression advantages of BEVS and biophysical similarity of *Sf*Basic-produced VLPs to HEK293-produced VLPs prompted further investigation into suitability for use as a vaccine immunogen. Guinea pigs were vaccinated with purified VLPs produced by *Sf*Basic or a VLP standard produced by HEK293 in 10-fold dose escalations spanning from 0.01 to 10 μg VLPs per dose. Both purified VLP preparations were adjuvanted equivalently with Adju-Phos aluminum adjuvant. Guinea pigs were vaccinated on study day 0 and 14, and serum anti-CHIKV IgG concentrations and CHIKV 181/25 neutralizing titers were determined on study day 14 (pre-boost) and 21 to compare the immunogenicity of an *Sf*Basic-derived VLP vaccine to the HEK293-derived VLP standard vaccine.

**Figure 7:**
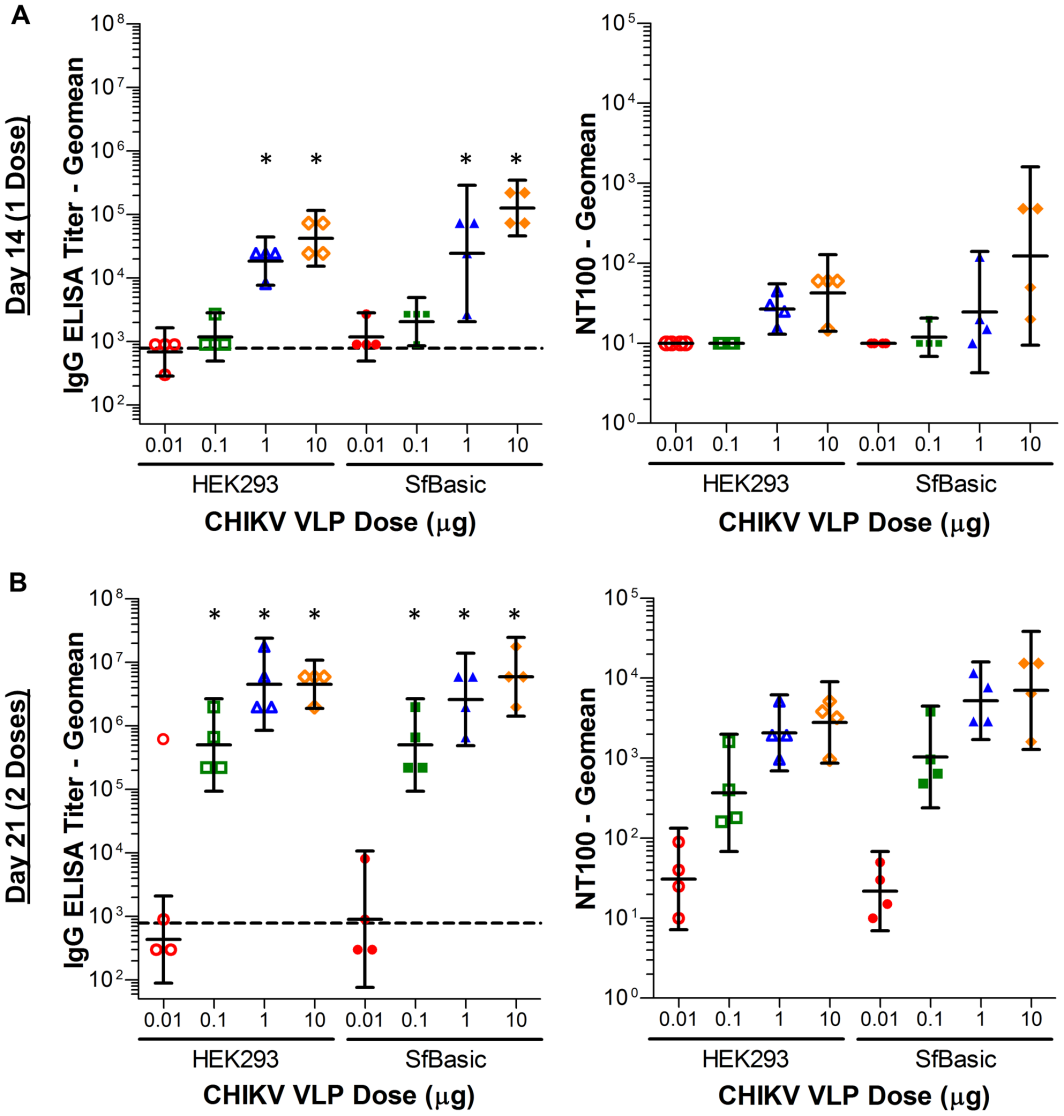
Immunogenicity assay titers from guinea pig sera after vaccination with adjuvanted *Sf*Basic-derived and HEK293-derived CHIKV VLPs. (**A**) Anti-CHIKV IgG geomean titer and CHIKV 181/25 geomean neutralizing titer (NT100) at study day 14 (14 days after first dose). (**B**) Anti-CHIKV IgG geomean titer and CHIKV 181/25 geomean neutralizing titer (NT100) at study day 21 (7 days after second dose). Geomean IgG ELISA background from pre-vaccination sera is indicated by a dashed line. Error bars represent 95% confidence intervals (N = 4 animals per group), and asterisks indicate a statistically significant increase in titer over background (p<0.05).

As shown in Figure 7, anti-CHIKV IgG ELISA titers were significantly elevated above pre-vaccination background in both the 1 and 10 μg dose groups after only 1 vaccination (Kruskal-Wallis test with Dunn's post-test, p<0.05), but CHIKV 181/25 neutralization titers were not substantially increased. After 2 vaccinations, neutralizing titers increased steadily with dose from 0.01 to 1 μg before stabilizing. These trends suggest a minimum VLP dose in the range of 1 to 10 μg, which is consistent with the ongoing Phase I clinical trial dose escalation starting point of 10 μg (ClinicalTrials.gov Identifier NCT01489358). Both anti-CHIKV IgG ELISA titers and neutralization titers for the *Sf*Basic derived VLP vaccine were not significantly different from the HEK293-derived VLP standard vaccine after 1 or 2 doses (Kruskal-Wallis test with Dunn's post-test for dose-matched groups). This overall similarity in immunogenicity confirmed that recombinant CHIKV VLPs produced from baculovirus infected *Sf*Basic cells could be a viable alternative vaccine candidate to VLPs produced by transient gene expression in mammalian cells.

## Conclusions

CHIKV strain 37997 VLPs were successfully produced from *Sf*21 insect cells by infection with a recombinant baculovirus vector at elevated culture pH (6.6–6.8) relative to standard insect cell culture pH (6.0–6.4). The necessary pH for enhanced VLP production was outside the optimal range for *Sf*21, so a custom high pH culture medium was developed and the *Sf*21 host cell line was selected and adapted by continuous exposure to increasing pH for several months. pH stress-induced adaptation led to the establishment of a novel insect cell line variant (*Sf*Basic) that is capable of growing normally and robustly at pH 6.8–7.2, where the parental *Sf*21 grows very slowly and inconsistently. The newly established pH-adapted cell line demonstrated an 11-fold increase in CHIKV VLP production over the maximum yield achieved by *Sf*21, producing approximately 28 mg/L culture on average. CHIKV VLPs were purified from the culture supernatant in a chromatographic process and confirmed to be structurally similar to an HEK293-derived CHIKV VLP standard comparable to the VLP vaccine currently under evaluation in a Phase 1 clinical trial. There was no significant difference in immunogenicity between *Sf*Basic-derived and VLP standard preparations as determined by total anti-CHIKV IgG or neutralizing titers in the serum of vaccinated guinea pigs. The cell line and process described herein could thus be used to produce a CHIKV VLP vaccine, and may be preferable to transient gene expression in mammalian cells for industrial vaccine manufacturing. The novel adaptation of *Sf*21 to grow robustly and produce high levels of recombinant VLPs in the pH range typical of mammalian cell culture may also have applications for the production of other pH-sensitive recombinant protein or VLP targets.
